# New prediction method of horizontal principal stress of deep tight reservoirs

**DOI:** 10.1038/s41598-022-16954-1

**Published:** 2022-07-28

**Authors:** Xinxin Fang, Jianbin Zhang, Tao Liu, Zhen Zhang, Fengling Li

**Affiliations:** 1 China Coal Research Institute, Beijing, 100013 China; 2 China Coal Technology & Engineering Group Xian Institute, Xian, 710077 Shanxi China; 3 Oilfield Technology Service Company of Xinjiang Oilfield Company, PetroChina, Karamay, Xinjiang, 834000 China; 4 Karamay Hongshan Oilfield Co., Ltd of Xinjiang Oilfield Company, PetroChina, Karamay, Xinjiang, 834000 China

**Keywords:** Environmental sciences, Natural hazards, Solid Earth sciences, Energy science and technology, Engineering

## Abstract

As the tight reservoir of Lucaogou Formation in Jimsar sag is characterized as large burial depth and poor physical properties, hydraulic fracturing technology is greatly needed to increase the production. Firstly, based on elastic theory, the borehole displacement formula under stress is derived, then the borehole quasi-ellipse characteristic model is proposed, on this basis, the in-situ stress prediction model based on borehole deformation is constructed. Secondly, the force analysis of diamond bit during drilling is performed, and the continuous prediction formula of elastic modulus based on weight on bit (WOB) and torque parameters is derived. Combined with these two steps, a new model of continuous in-situ stress prediction based on borehole deformation and drilling parameters is established. Compared with the values measured by the acoustic emission method in the laboratory, the deviation between the predicted in-situ stress values and those measured by the acoustic emission method is less than 4%, which meets the accuracy requirements of the fracturing in the oil field. The prediction results show that the minimum horizontal principal stress of Lucaogou Formation is 51.1–62.7 MPa, and the maximum horizontal principal stress is 58.9–69.1 MPa, respectively. The maximum horizontal principal stress direction is NW–SE direction with an angle of about 159°. The results show that the fracture direction in the study area is in accordance with the present in-situ stress direction, which is beneficial to the secondary reconstruction of natural fractures and keeps good opening property of fractures. This study provides a theoretical basis for in-situ stress prediction and compressibility evaluation of tight reservoir.

## Introduction

In-situ stress measurement and prediction are of great practical and economic significance in the exploration and development of oil and gas fields^1–3^. In-situ stress is one of the driving forces for oil and gas migration and accumulation. The fractures, faults and tectonic formed under in-situ stress are one of the channels and places for oil and gas migration and accumulation^4–7^. Presently, in-situ stress field affects and controls the dynamic changes of oil, gas and water in the development of oil and gas fields. By analyzing the relationship between in-situ stress and fractures, we can study the law of oil and gas migration and accumulation, and search for petroliferous basins^8–11^. Based on the distribution characteristics of in-situ stress and reservoir lithologic parameters, we cannot only predict the law of fracture propagation, but also provide a basis for making reasonable oil and gas development plans. In addition, the formation pressure profile (fracture pressure and collapse pressure) can be established to predict the borehole stability of oil drilling engineering. In-situ stress is the basic data of oilfield development plan design, hydraulic fracturing fracture propagation law analysis, formation fracture pressure and formation collapse pressure prediction^12–14^. Without an accurate continuous in-situ stress profile, any simulation calculation of fracture geometry will be non-meaningful. Therefore, it is of great significance to obtain accurate crustal stress data for oil and gas exploration and development.

At present, the commonly used in-situ stress measurement methods include hydraulic fracturing method, acoustic emission method, borehole caving method, FMI and CBIL imaging logging method, seismic velocities method, as well as remote sensing technology^15–20^. Although hydraulic fracturing method can measure in-situ stress in deep strata, it must be assumed that one of the principal stress directions is consistent with borehole axial. This method is more suitable for homogeneous and isotropic strata. Acoustic emission method based on Kaiser effect can accurately measure in-situ stress of rock, but it cannot obtain continuous in-situ stress profile due to the limited number of cores and the need to complete in laboratory conditions^21,22^. The borehole caving method is mainly based on the borehole wall caving imaging to obtain the width of the caving during the drilling process to predict the magnitude of in-situ stress. The long axis direction of elliptical borehole formed by borehole caving corresponds to the direction of minimum principal stress, but the number of borehole caving is limited during drilling. FMI and CBIL imaging logging methods mainly image induced fractures and tensile fractures formed by drilling, and the fracture direction is consistent with the direction of maximum principal stress^23–25^. This method can accurately predict the direction of in-situ stress, but it cannot achieve quantitative prediction of in-situ stress. Moreover, this method is expensive, and it is not easy to be popularized in the whole oilfield. The seismic wave velocity method and remote sensing technology are easily disturbed by external signals and the energy of effective signals is dissipated.

The author takes the tight reservoir of Jimsar Sag in Jungar Basin as an example. This area is faced with many problems, such as tightness of reservoir and poor development. Most reservoirs need to be fractured. Accurate prediction of in-situ stress characteristics of reservoir is a crucial link to optimize fracturing scheme design and promote efficient development. In this paper, the continuous in-situ stress measurement prediction model based on the integration of drilling and measuring is established combined with the acoustic emission experiments and theoretical derivation:

Firstly, based on the theory of elastic and classical rock mechanics, deriving the quasi-ellipse shape of a circular hole formed under the action of plane two-dimensional stress, in addition establishing the relationship between horizontal principal stress and quasi-ellipse geometric parameters; Secondly, the measurement methods and testing techniques of quasi-ellipse geometric parameters are studied to obtain the borehole shape parameters which reflect the in-situ stress indirectly; Finally, based on the continuous drilling parameters in the process of drilling, the elastic parameters needed for borehole shape to calculate in-situ stress are deduced, and the dynamic in-situ stress prediction model is established. The morphological characteristics of natural fractures and compressibility in the study area are evaluated by the stress profile predicted with the new method, which provides a reference for hydraulic fracturing of tight reservoir of Lucaogou Formation. The method proposed in this paper provides a new idea and method to solve the problem of continuous in-situ stress measurement in deep complex environment, realizes the continuous in-situ stress prediction, and solves the problems of current in-situ stress data, such as time-consuming measurement, high cost, data discontinuity and limited depth.

## Geologic setting

**Figure 1:**
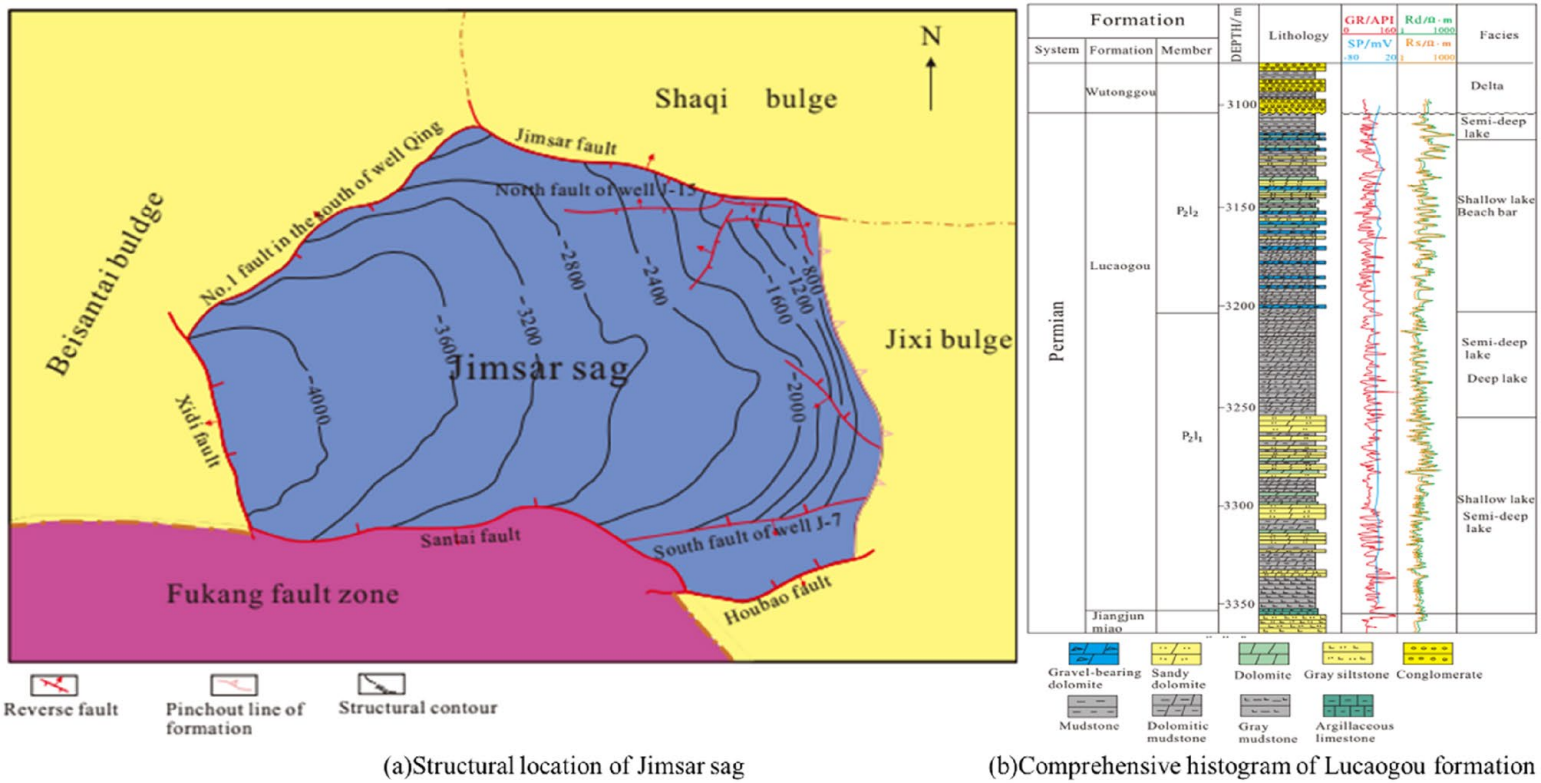
Comprehensive histogram of structural location and Lucaogou formation in Jimsar Sag.

As can be seen from Fig. 1a, Jimsar sag is located in the southeastern margin of the eastern Jungar Basin. It is a secondary depression on the eastern uplift of the first-level tectonic unit of the basin with the length of about 60 km from east to west and width of about 50 km from north to south, characterized as dustpan shape, covering an area of about 1300km^2^^26–28^. Thrust faults oriented to sag exist in the south, west and north, bounded by Santai fault in the south, Xidi fault in the west and No. 1 fault in the South of Qing1 Well in the west, Guxi uplift in the east, Jimsar fault and Shaqi uplift in the northeast. The strata in the study area are composed of Carboniferous, Permian, Triassic, Jurassic, Cretaceous, Paleogene and Neogene. Lucaogou Formation is divided into two sections longitudinally (Fig. 1b), and lithology mainly includes dolomitic sandstone, dolomitic mudstone, sandy dolomite, argillaceous dolomite and mudstone. The reservoir porosity of Lucaogou Formation is 6%-22.8%, with an average of 10.3%, and the permeability is 0.001mD-0.273mD, with an average of 0.01mD and a permeability less than 0.1mD, which is a typical tight reservoir^29–31^. During the Middle Permian, Jimsar area was located in shallow saline lake basin with arid climate, and the regional volcanic activity was strong. Mantle-derived hydrothermal activity developed in the lake basin, which was conducive to the formation of dolomite mineral crystals. Therefore, the middle Permian stratum was dominated by dolomite. As the lithology is dominated by dolomite, dolomitic sandstone as well as dolomitic mudstone, the borehole deformation was less affected by hydration of drilling mud. The formation is stable and not easy to collapse. The borehole deformation of Lucaogou Formation is mainly produced by the unbalance of horizontal principal stress.

## Methods and principles

### Quantitative relationship between borehole deformation and in-situ stress

#### Relation between shaft wall displacement and in-situ stress

In-situ stress measurement is usually based on plane stress, and the well hole is equivalent to a circular hole in an infinite thin plate with radius *a*. It is assumed that the rock mass is an infinite elastic body, the borehole is subjected to far-field stress, the elastic modulus of the rock mass is E, and the Poisson's ratio is *V*. Assuming that the tension is positive and the pressure is negative, the stress in the plane coordinate system can be expressed as:

1 $$\left\{\sigma \right\}=\left\{{\sigma }_{H},{\sigma }_{h}\right\}$$

**Figure 2:**
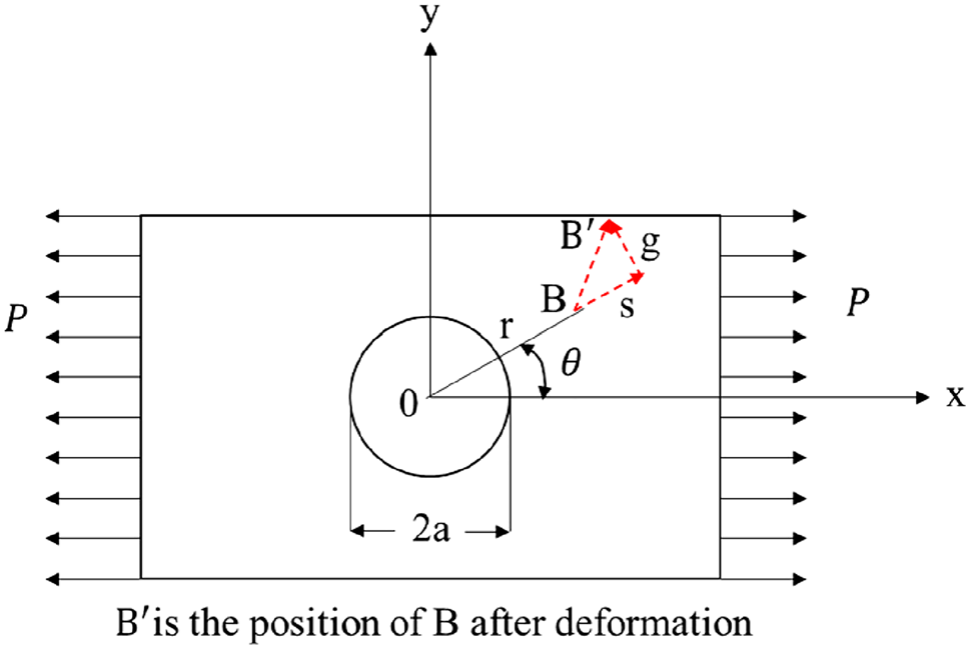
Displacement near borehole.

According to the basic theories of elasticity and rock mechanics, the radial displacement **s** and tangential displacement ***g*** of any point ***B*** on the shaft wall (Fig. 2) under the action of unidirectional stress ***P*** can be expressed as follows:

2 $$\left. {\begin{array}{*{20}c} {s\left| {_{r = a} } \right. = \frac{aP}{E}\left[ {1 + 2\cos \left( {2\theta } \right)} \right]} \\ {g\left| {_{r = a} } \right. = \frac{ - 2aP}{E}\sin \left( {2\theta } \right)} \\ \end{array} } \right\}$$

**Figure 3:**
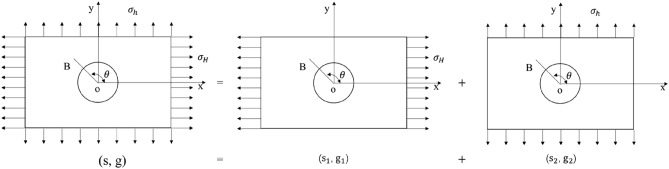
Superposition diagram of shaft wall displacement.

When subjected to bidirectional stress ${\sigma }_{H}$ and ${\sigma }_{h}$ in the x and y directions, the radial displacement on the well wall can be obtained by the superposition method, as shown in the Fig. 3.

It is assumed that s_1_ is the radial displacement of the shaft wall at point B when the stress is only in the *X* direction, and s_2_ is the radial displacement of the rock mass at point B when the stress is only in the *Y* direction.

According to Eq. (2), it can be obtained:

3 $$\left. \begin{gathered} s_{1} = \frac{{a\sigma_{H} }}{E}\left[ {1 + \cos \left( {2\theta } \right)} \right] \hfill \\ g_{1} = \frac{{ - 2a\sigma_{H} }}{E}\sin \left( {2\theta } \right) \hfill \\ \end{gathered} \right\}$$

4 $$\left. \begin{gathered} s_{2} = \frac{{a\sigma_{h} }}{E}\left[ {1 + \cos \left( {2\alpha } \right)} \right] \hfill \\ g_{2} = \frac{{ - 2a\sigma_{h} }}{E}\sin \left( {2\alpha } \right) \hfill \\ \end{gathered} \right\}$$

where: a is the circumferential radius of point ***B***; θ and α are the included angles between the radial direction of point ***B*** and the positive x-axis and y-axis respectively, and the relationship between them is as follows:

5 $$\alpha =\theta -90^\circ$$

Therefore, it can be obtained as following equation when substituting Eq. (5) into Eq. (4):

6 $$\left. \begin{gathered} s_{2} = \frac{{a\sigma_{h} }}{E}\left[ {1 - \cos \left( {2\theta } \right)} \right] \hfill \\ g_{2} = \frac{{2a\sigma_{h} }}{E}\sin \left( {2\theta } \right) \hfill \\ \end{gathered} \right\}$$

According to the superposition principle, the shaft wall displacement under the combined action of the two external forces is equal to the sum of the shaft wall displacement caused by one-way stress alone, namely:

7 $$\left. \begin{gathered} s = s_{1} + s_{2} = \frac{a}{E}\left[ {\sigma_{H} + \sigma_{h} + 2\left( {\sigma_{H} - \sigma_{h} } \right)\cos \left( {2\theta } \right)} \right] \hfill \\ g = g_{1} + g_{2} = - \frac{2a}{E}\left( {\sigma_{H} - \sigma_{h} } \right)\sin \left( {2\theta } \right) \hfill \\ \end{gathered} \right\}$$

Equation (7) is the basic formula for measuring in-situ stress by using borehole deformation based on plane stress state, which reflects the displacement change of any point of borehole wall under stress.

#### Shape characteristics of quasi-elliptic borehole under in-situ stress

**Figure 4:**
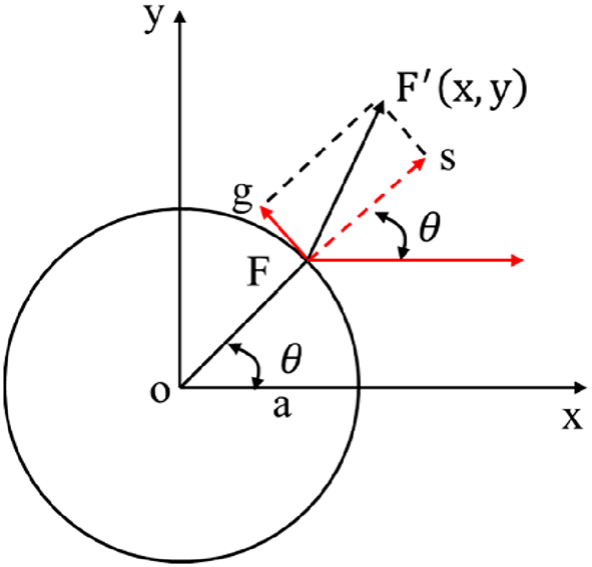
Variation at any point of the circular hole.

According to the analytical solution in the previous section, the displacement of any point ***F*** on the shaft wall can be expressed by Eq. (7). Assuming that point ***F*** deforms to point ${{\varvec{F}}}^{\boldsymbol{^{\prime}}}$ at coordinates (x, y), as shown in Fig. 4, then

8 $$\left. \begin{gathered} x = a * \cos \theta + s * \cos \theta - g * \sin \theta \hfill \\ y = a * \cos \theta + s * \sin \theta + g * \cos \theta \hfill \\ \end{gathered} \right\}$$

Substituting Eq. (7) into Eq. (8), after simplification, it could be obtained that:

9 $$\left. {\begin{array}{*{20}c} {x = a\left[ {1 + \left( {3\sigma_{H} - \sigma_{h} } \right)/E} \right]\cos \theta } \\ {y = a\left[ {1 + \left( {3\sigma_{h} - \sigma_{H} } \right)/E} \right]\sin \theta } \\ \end{array} } \right\}$$

Let:

10 $$\left. \begin{gathered} A_{0} = a\left[ {1 + \left( {3\sigma_{H} - \sigma_{h} } \right)/E} \right] \hfill \\ B_{0} = a\left[ {1 + \left( {3\sigma_{h} - \sigma_{H} } \right)/E} \right] \hfill \\ \end{gathered} \right\}$$

Then Eq. (9) can be expressed as:

11 $$\left. {\begin{array}{*{20}c} {x = A_{0} \cos \theta } \\ {y = B_{0} \sin \theta } \\ \end{array} } \right\}$$

Equation (11) meeting the following standard equation of ellipse:

12 $$\frac{{x^{2} }}{{A_{0}^{2} }} + \frac{{y^{2} }}{{B_{0}^{2} }} = \cos^{2} \theta + \sin^{2} \theta = 1$$

However, due to the difference in elastic modulus of rock at different depths, *A*_*0*_ and *B*_*0*_ vary with the burial depth. Therefore, the geometric form of circular hole deformation under in-situ stress can be approximated as ellipse, namely, quasi-elliptic structure. Assuming that *A* and *B* are respectively the lengths of major and minor semi-axes of quasi-ellipse, we can solve ${\sigma }_{H}$ and ${\sigma }_{h}$ by Eq. (10) as follows:

13 $$\left. {\begin{array}{*{20}c} {\sigma_{H} = \frac{{3A_{0} + B_{0} - 4a}}{8a}E} \\ {\sigma_{h} = \frac{{A_{0} + 3B_{0} - 4a}}{8a}E} \\ \end{array} } \right\}$$

The maximum horizontal principal stress ${\sigma }_{H}$ and minimum horizontal principal stress ${\sigma }_{h}$ can be calculated by measuring the length of long and short semi-axis (*A, B*) of quasi-ellipse.

**Figure 5:**
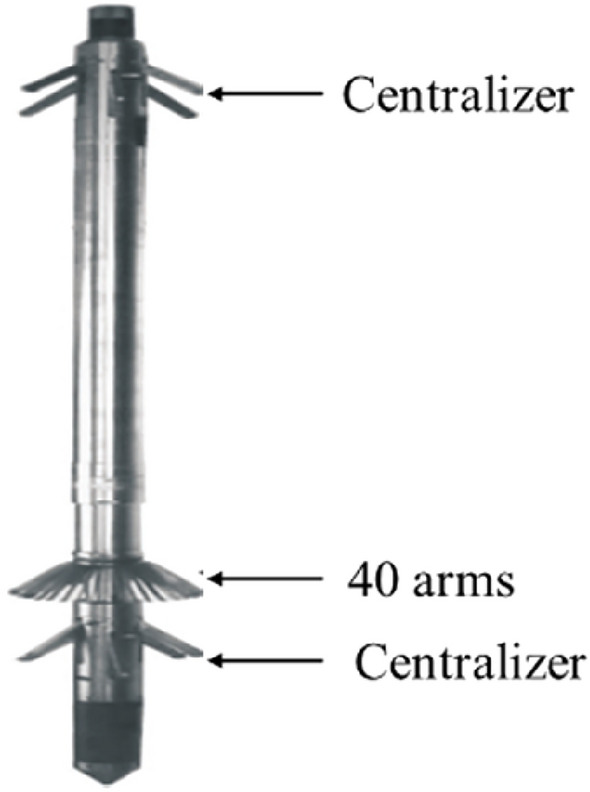
40-arm Caliper Imaging Logging tool.

The values of *A* and *B* can be obtained by the 40-arm caliper imaging logging tool. The 40-arm caliper logging tool outputs 40 independent diameter curves. The maximum diameter, minimum diameter, and average diameter curves for each cross section can be calculated and output from these 40 diameter curves (Fig. 5).

#### Optimization of long and short axis of quasi-ellipse wellbore

The long and short axes of the quasi-ellipse correspond to the maximum and minimum diameters respectively. The maximum and minimum output caliper values were obtained by optimizing 40 caliper curves. Assuming that 40 data at a certain depth with the instrument axis as the center of the circle are respectively *r*_*i*_ (*i* = *1,2,3…,40*), and the rectangular coordinates of the point where the caliper tool axis is on the section are (0,0). Taking the borehole direction corresponding to the first data point r_1_ as the positive *x*-axis, and the borehole direction corresponding to the eleventh data point r_11_ as the positive *y*-axis. In this plane rectangular coordinate system, the measurement value of the i-th arm in the *x*-axis and *y*-axis directions are set to *x*_*i*_*, y*_*i*_, then:

$${x}_{i}={r}_{i}\mathrm{cos}\frac{2\pi \left(i-1\right)}{40},{y}_{i}={r}_{i}\mathrm{sin}\frac{2\pi \left(i-1\right)}{40}\left(i=\mathrm{1,2},\cdots ,40\right)$$

Supposing the standard diameter of the wellhole is *R*, (*x, y*) is the coordinate of any point in the section, expressed as:

14 $${L}_{i}\left(x,y\right)=\left\{\begin{array}{c}1,\mathrm{if }\sqrt{{\left(\mathrm{x}-{\mathrm{x}}_{\mathrm{i}}\right)}^{2}+{\left(\mathrm{y}-{\mathrm{y}}_{\mathrm{i}}\right)}^{2}}=\mathrm{R}\\ 0,\mathrm{if}\sqrt{{\left(\mathrm{x}-{\mathrm{x}}_{\mathrm{i}}\right)}^{2}+{\left(\mathrm{y}-{\mathrm{y}}_{\mathrm{i}}\right)}^{2}}\ne \mathrm{R}\end{array}\right.$$

15 $$L\left(x,y\right)=\sum_{i=1}^{40}{L}_{i}\left(x,y\right)$$

If $\left|\sqrt{{\left(\mathrm{x}-{\mathrm{x}}_{\mathrm{i}}\right)}^{2}+{\left(\mathrm{y}-{\mathrm{y}}_{\mathrm{i}}\right)}^{2}}-R\right|<\varepsilon$,taking ${L}_{i}\left(x,y\right)$=1, otherwise ${L}_{i}\left(x,y\right)$=0, where $\varepsilon$ is the precision, which varies according to the resolution of the caliper instrument.

If the borehole is not deformed, then:

16 $${x}_{i}^{2}+{y}_{i}^{2}={R}^{2}\left(i=\mathrm{1,2},\cdots ,40\right)$$

If the borehole is deformed, as the deformed section is no longer a standard circle, we define (*a, b*) meeting $L\left(a,b\right)=\underset{\left(x,y\right)}{\mathrm{max}}L\left(x,y\right)$ as the approximate center coordinate of the section, which is reasonable for the problem we study. According to the characteristics of objective function L (*x, y*), mode search method in optimization technology is adopted for numerical solution of (*a, b*), and the initial point of iteration in the algorithm is selected in the following form:

17 $${x}_{i}^{0}=\frac{1}{40}\sum_{i=1}^{40}{x}_{i},{y}_{i}^{0}=\frac{1}{40}\sum_{i=1}^{40}{y}_{i}$$

Although the pattern search method is a local search technique, the local optimum should be the global optimum when the initial point is selected in the above way. After a lot of trial calculations, this point has been confirmed.

When the values of *a* and *b* are determined, 2 $\underset{i}{\mathrm{min}}\sqrt{{\left({x}_{i}-a\right)}^{2}+{\left({y}_{i}-b\right)}^{2}}$ is the minimum inner diameter of the deformed section, 2 $\underset{i}{\mathrm{max}}\sqrt{{\left({x}_{i}-a\right)}^{2}+{\left({y}_{i}-b\right)}^{2}}$ is the maximum inner diameter of the deformed section.

### Quantitative relationship between drilling parameters and rock mechanics parameters

When a diamond coring bit is rotating through rock, if the bit is drilling at constant rotation speed and constant drilling rate, the bit will receive a matched response pressure and response torque. For the same rock, the pressure and torque are constant. In contrast, if the bit is drilling through rock at constant pressure and rotation speed, the bit will receive a matched response rate and torque, which are also constant for the same rock. In other words, the drilling parameters and response parameters are matched.

**Figure 6:**
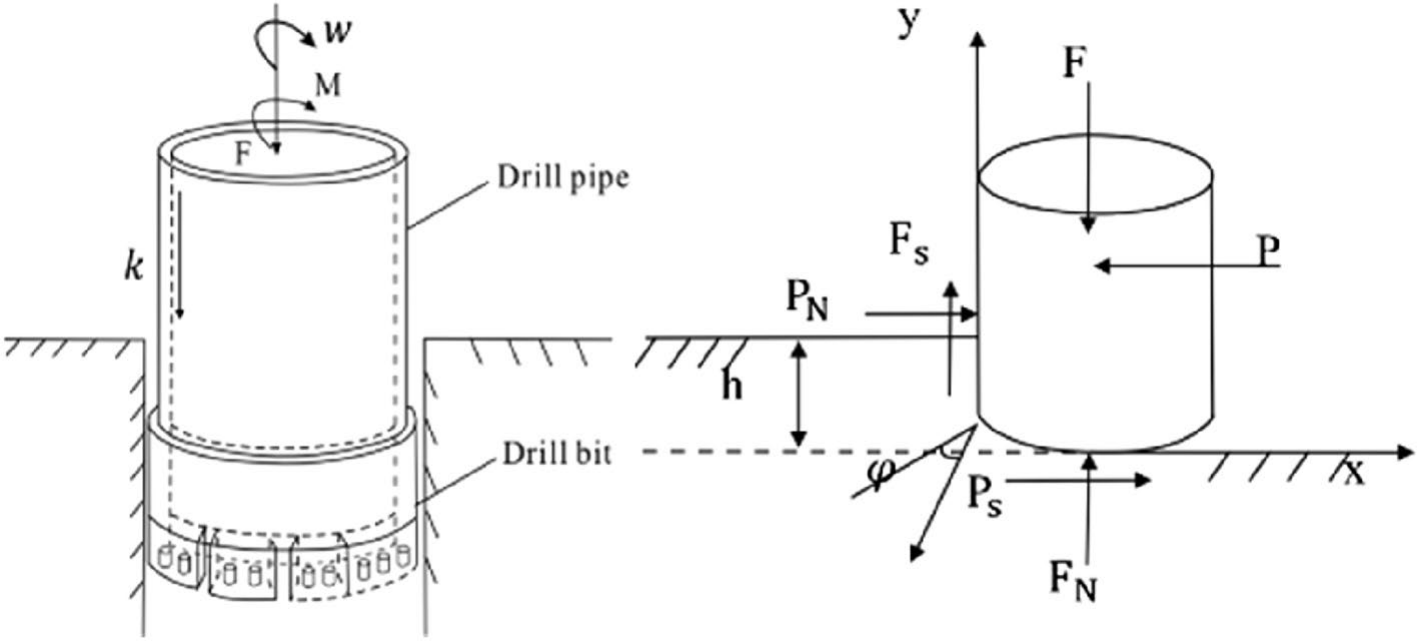
Stress diagram of single diamond.

In order to obtain the elastic modulus of rock, the stress of diamond particles is analyzed firstly. In addition to the vertical static pressure F from the static loading device and the horizontal cutting force *P* provided by the rotary cutting device, diamond particles are also subjected to the rock's reaction forces *F*_*N*_ and *P*_*N*_. Friction *P*_*s*_ is distributed on the friction surface at the bottom of diamond particles and friction *F*_*s*_ is distributed at the friction surface at the front of diamond, as shown in Fig. 6. If the friction coefficient between the friction surface and the rock is $\mu$, then ${F}_{s}=\mu {P}_{N}$. According to the force balance conditions, it could be obtained:

18 $$\sum X=0 P-{P}_{N}-\mu {F}_{N}=0$$

19 $$\sum Y=0 F-{\mu P}_{N}-{F}_{N}=0$$

According to Eq. (18) and (19), it could be obtained:

20 $${F}_{N}=\frac{F-\mu P}{1-{\mu }^{2}}$$

Under the action of propulsive force, the drilling bit exerts pressure and cutting force on the rock mass at the bottom of the drilling hole, which makes the rock mass in a certain area cut and squeeze failure. The diamond particles were assumed to be rigid cylinders with radius *R*, and the rock was assumed to be elastomers, which were pressed into depth *h* under the action of external forces. According to the analysis of Boussinesq. J. V problem, the stress distribution of the surface can be regarded as the action of strip regional distribution force:

21 $${f}_{0}={F}_{0}{\left(1-\frac{{r}^{2}}{{R}^{2}}\right)}^{-1/2}$$

where: *F*_*0*_ is the contact pressure. According to elasticity, the displacement of rock surface points along the *Z*-axis is

22 $${u}_{z}=\frac{1-{k}^{2}\pi {F}_{0}R}{E}=h$$

Thus, the central compressive stress and contact pressure are obtained:

23 $${F}_{0}=\frac{Eh}{\left(1-{k}^{2}\right)R\pi }$$

24 $${F}_{N}=2\pi {F}_{0}{R}^{2}=\frac{2ERh}{\left(1-{k}^{2}\right)}$$

Then the elastic modulus is as following:

25 $$E=\frac{{F}_{N}\left(1-{k}^{2}\right)}{2hR}$$

Substituting Eq. (25) into (24), it could be obtained:

26 $$E=\frac{\left(F-\mu P\right)\left(1-{k}^{2}\right)}{2hR\left(1-{\mu }^{2}\right)}$$

where: $P=\frac{3M}{2\pi {n}_{0}\left({D}^{3}-{d}^{3}\right)}$,$h=\frac{60v}{{N}_{i}w}$, $\mu =\frac{2M}{P\left(d+D\right)}$. $\mu$- friction coefficient; *D*-outer diameter of the bit; *d*-the inner diameter of bit; *M*-torque; *R*-radius of diamond particles. *F*-weight on bit. *n*_*0*_-number of blades per unit area; *k*-drilling speed; *N*_*i*_-the row number of diamond particles on the bottom of the bit; $w$-rotation speed.

### Dynamic continuous measurement model of in-situ stress

Based on the above quantitative relationship model between borehole shape and in-situ stress and theoretical formula derivation of rock elastic parameters based on drilling parameters, a continuous in-situ stress prediction model based on borehole deformation and measurable drilling parameters was obtained:

27 $$\left.\begin{array}{c}{\sigma }_{H}=\frac{\left(3{A}_{0}+{B}_{0}-4a\right) \cdot \left(F-\mu P\right) \cdot \left(1-{k}^{2}\right)}{16ahR \cdot \left(1-{\mu }^{2}\right)}\\ {\sigma }_{h}=\frac{\left({A}_{0}+{3B}_{0}-4a\right) \cdot \left(F-\mu P\right) \cdot \left(1-{k}^{2}\right)}{16ahR \cdot \left(1-{\mu }^{2}\right)}\end{array}\right\}$$

## Experiments

### Samples

**Figure 7:**
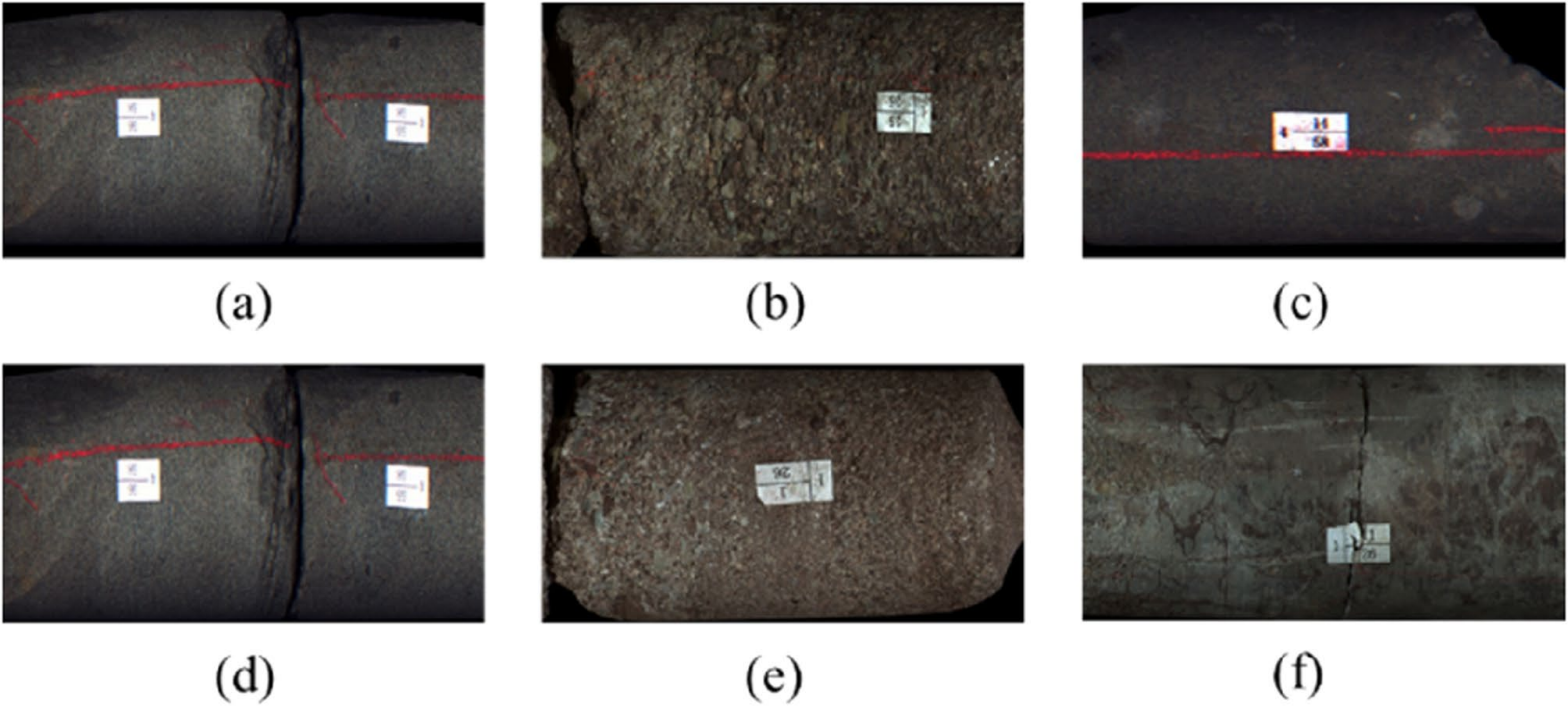
Core samples. (**a**) Siltstone; (**b**) Calcite mudstone; (**c**) Dolomite; (**d**) Dolomitic sandstone; (**e**) Gritstone; (**f**) Dolomite mudstone.

**Figure 8:**
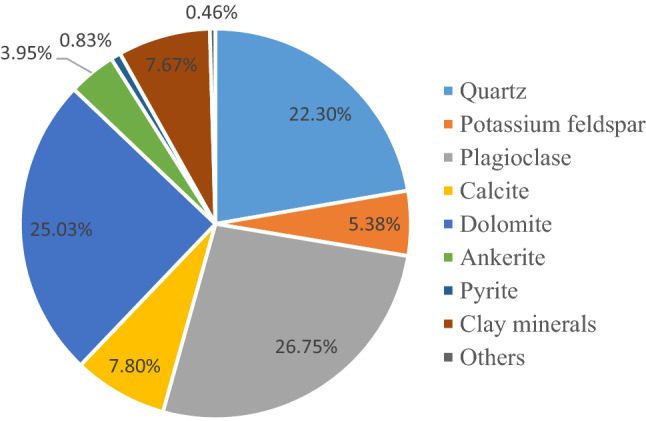
Mineral composition of rock sample.

The cores in the experiment are from well J-37, J-21 and J-25 of the Lucaogou Formation in Jimusar Sag (Fig. 7). The lithology is siltstone, calcite mudstone, dolomitic mudstone and argillaceous siltstone. The size of the rock sample is a small cylinder with a diameter of 2.5 cm and a height of 5 cm. Based on core observation, thin section identification, scanning electron microscopy and X-ray diffraction analysis, it can be concluded that the fine-grained mixed sedimentary rocks of Lucaogou Formation in Jimusar Sag are dominated by felsic minerals and carbonate minerals, with low content of clay minerals and a small amount of pyrite and zeolite. The content of felsic minerals is 6.9% ~ 94.6%, with an average content of 54.26%, mainly quartz and plagioclase. Carbonate minerals include dolomite and calcite, ranging from 0% to 84.4%, with an average of 36.78%, and dolomite is dominant, with an average of 28.98%. The relative content of clay minerals is generally low, mainly less than 10%, and rarely more than 30%, with an average content of about 7.67% (Fig. 8).

### Experiment equipment

**Figure 9:**
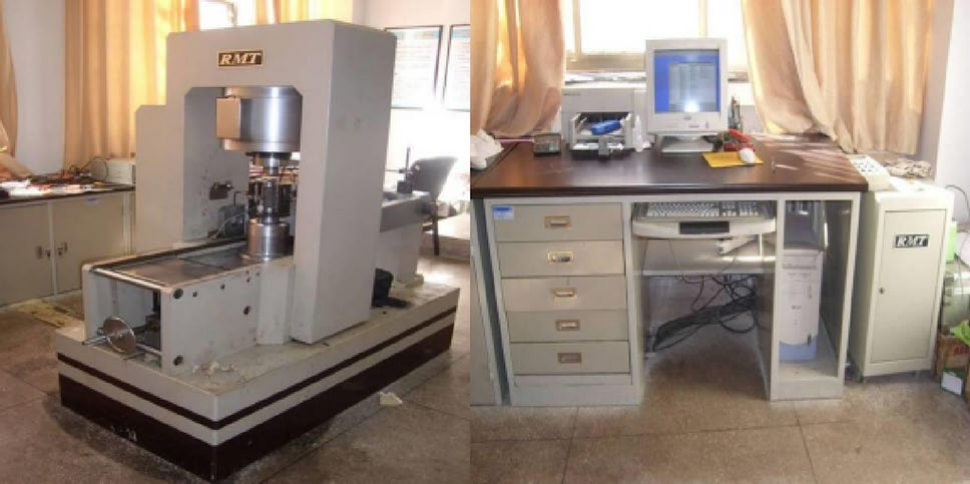
Rock mechanics test system and acoustic emission system.

RMT-100B rock mechanics servo testing machine and the Micro-II Express acoustic emission monitoring system produced by American Physical Acoustics Company are adopted to measure the in-situ stress of rock core indoor. The test system is shown in Fig. 9. During the test, the sampling frequency of the acoustic emission system was set to 3 MHz, and the threshold value was set 40 dB. In the experiment, 6 sensors were distributed in 3 levels, and they were coupled with butter. At the same time, rigid pads matching the diameter of the rock sample were added to both ends of the sample to reduce the impact of end-face friction on the test results.

### Data processing methods

**Figure 10:**
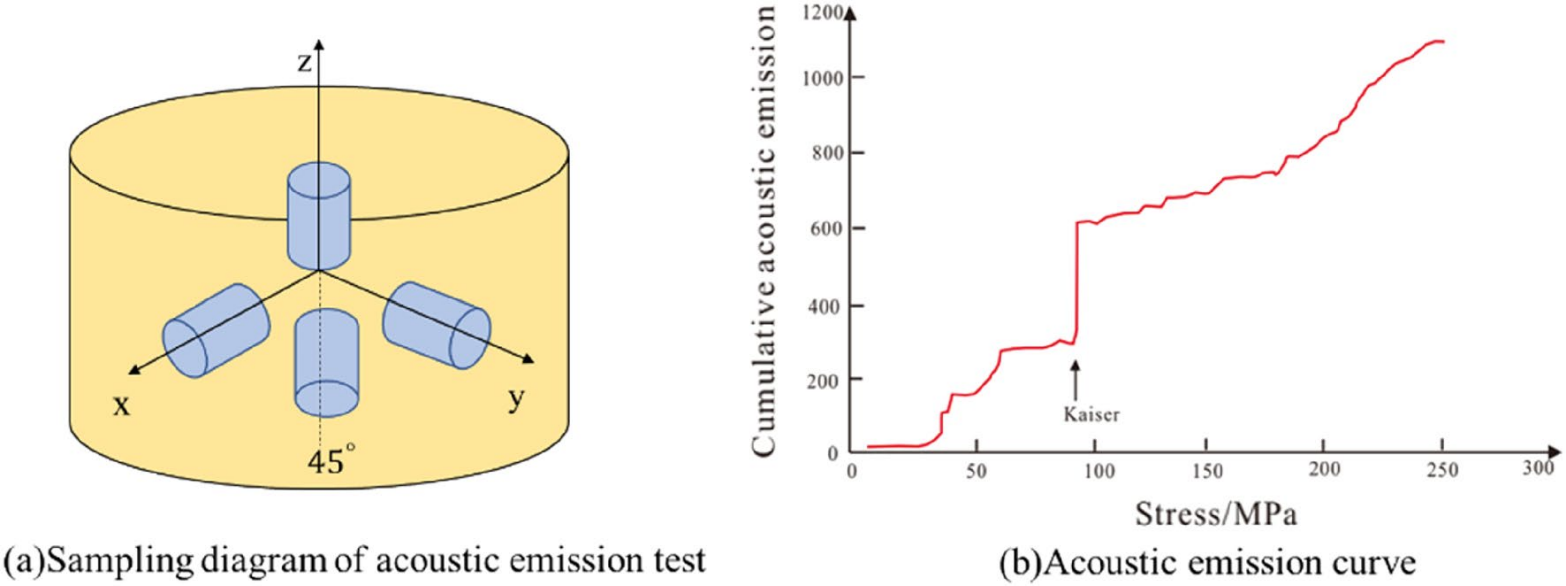
Determination of in-situ stress by acoustic emission.

As the rock has the memory of the previous load, when the rock is reloaded, if applied load is greater than the initial in-situ stress, the micro-cracks in the rock will rupture, resulting in the phenomenon of acoustic emission, namely, the Kaiser effect. The point where the emission frequency suddenly increases in the process loading is called the Kaiser point. Performing uniaxial compression test on samples taken from different directions (Fig. 10a), measuring the acoustic emission signal of the sample during the loading process, the values of different stress components are determined according to the sudden change point of the acoustic emission signal (Fig. 10b). The plane stress calculation formula based on elasticity is used to obtain the horizontal principal stress value (Eq. (28)).

28 $$\left\{ {\begin{array}{*{20}l} {\sigma _{v} = \sigma _{z} } \hfill \\ {\sigma _{H} = \frac{{\sigma _{x} + \sigma _{y} }}{2} + \sqrt {\left( {\frac{{\sigma _{x} - \sigma _{y} }}{2}} \right)^{2} + \left( {\sigma _{{xy}} - \frac{{\sigma _{x} + \sigma _{y} }}{2}} \right)^{2} } } \hfill \\ {\sigma _{h} = \frac{{\sigma _{x} + \sigma _{y} }}{2} - \sqrt {\left( {\frac{{\sigma _{x} - \sigma _{y} }}{2}} \right)^{2} + \left( {\sigma _{{xy}} - \frac{{\sigma _{x} + \sigma _{y} }}{2}} \right)^{2} } } \hfill \\ \end{array} } \right.$$

where: ${\sigma }_{z}$- the stress of the sample in the z direction; ${\sigma }_{x}$-the stress component of the sample in the x direction; ${\sigma }_{xy}$-the stress component of the sample in the 45° direction; ${\sigma }_{y}$- the stress component of the y direction.

## Results and validation

### Prediction of in-situ stress

**Figure 11:**
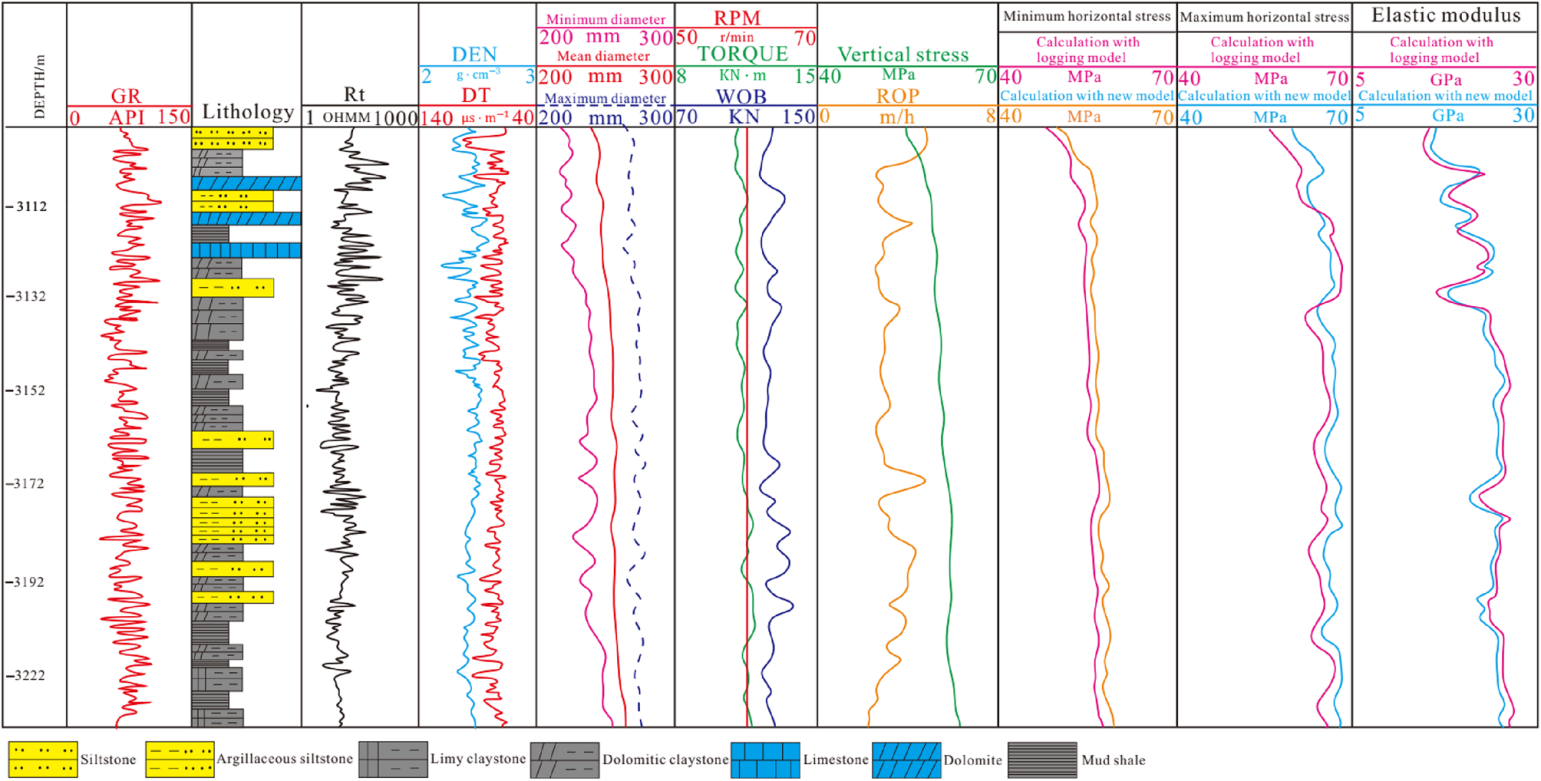
Prediction section of in-situ stress in well J-21.

**Figure 12:**
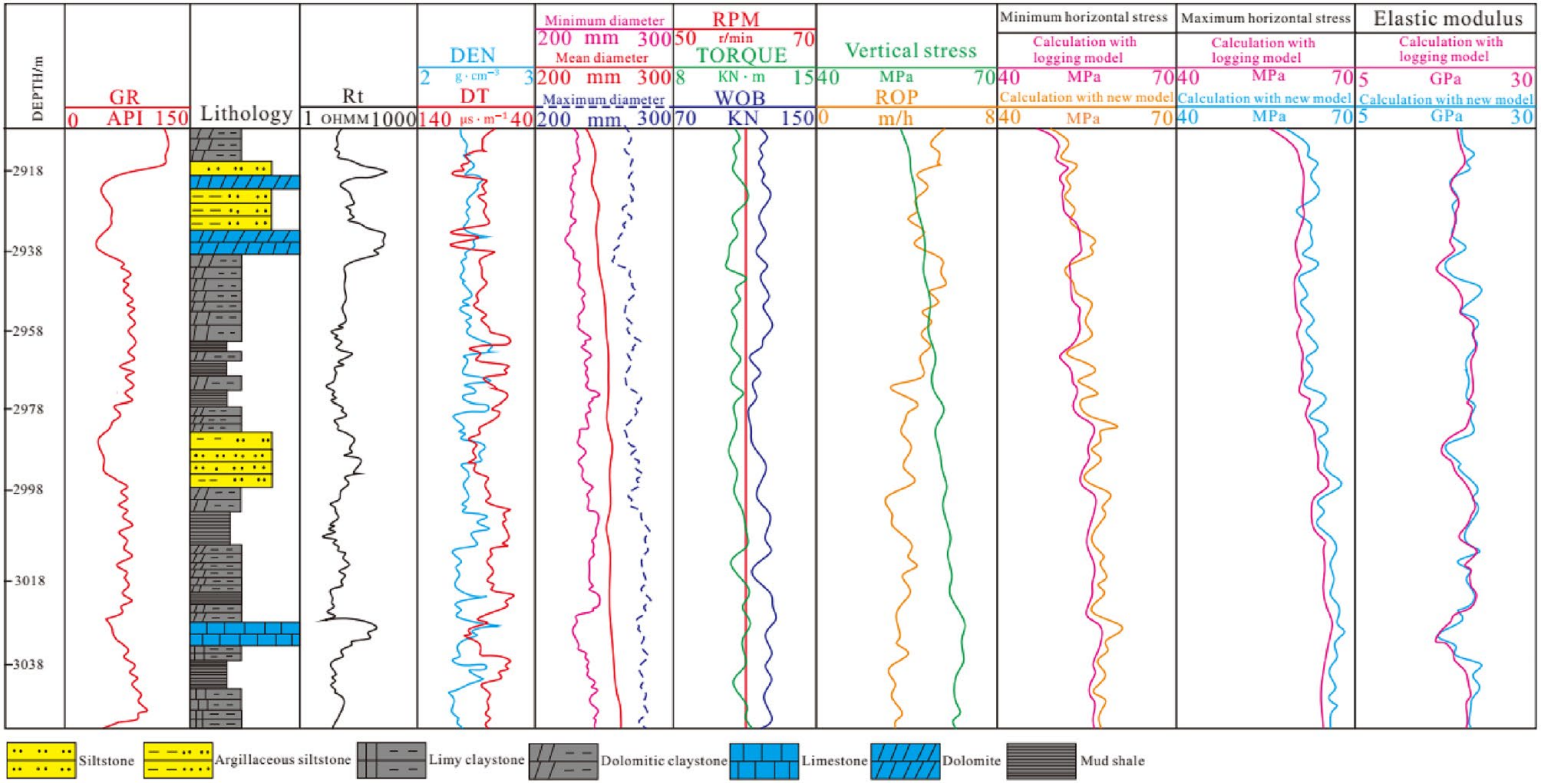
Prediction section of in-situ stress in well J-37.

Two wells J-21 and J-37 in Jimsar Sag were selected for testing. The new prediction model established in this paper and logging model (referred to ^32^) were applied to predict the in-situ stress of the Lucaogou Formation in these two wells, obtaining a continuous in-situ stress profile. According to the profile, it could be seen that the maximum horizontal principal stress of Lucaogou Formation is 58.9–69.1 MPa, and the minimum horizontal principal stress is 51.1–62.7 MPa. As it can be seen that (Fig. 11), the vertical stress generally increases with the increase of buried depth. Due to the complex lithology of tight reservoirs and the difference in rock mechanical parameters, the horizontal principal stress changes irregularly, but as a whole it still increases with the increase of depth. The trend of the increase is smaller than the vertical principal stress. As shown in Figs. 11 and 12, the average maximum and minimum horizontal principal stresses of siltstone and argillaceous siltstone are 55.4 MPa and 51.4 MPa, which are lower than those of mud shale, dolomitic mudstone and calcite mudstone. The average maximum and minimum horizontal principal stresses of these three rocks are 62.9 MPa and 54.6 MPa. The compressive strength of shale, dolomitic mudstone and calcite mudstone (8.7 MPa on average) is higher than that of siltstone and argillaceous siltstone (5.8 MPa on average) due to the strong compactness and brittleness of shale, dolomitic mudstone and calcite mudstone. According to the prediction results, the stress state in the studied area is Type III (${\upsigma }_{\mathrm{H}}>{\upsigma }_{\mathrm{V}}>{\upsigma }_{\mathrm{h}}$), called the strike-slip stress type, correspondingly, the fracture propagation direction is consistent with the direction of the maximum horizontal principal stress, but the fracture propagation ability is relatively weak in the longitudinal direction and relatively strong in the transverse direction.

### Validation

Comparing the predicted in-situ stress data by new model with the acoustic emission data (Table 1), the average error of the maximum horizontal principal stress is 3.21%, the average error of the minimum horizontal principal stress is 2.98%, and both errors are less than 4%, which can meet the accuracy requirements of the studied area and be generalized in the oil field. However, the corresponding error of prediction of maximum and minimum principal stress with logging methods are 5.30%,5.83% respectively, which are greater than that that of new model (Table 1). As it can be seen from Figs. 11 and 12 that, the variation of predicted profile of Yong’s modulus with logging model is approximately similar to that of new model. Comparing the prediction values of Young’ s modulus by logging model and new model with test values (Table 2), the errors of these two models are 8.91%,8.21% respectively. Though the errors of Young’s modulus are greater than that of horizontal principal stress, actually, the precision of predicted Young’s modulus meet the needs of production in the oilfield. Therefore, the predicted horizontal principal stress and Yong’s modulus are consistent with measurements in the experiments and conform to the actual geological characteristics, moreover it is appliable in the development of deep tight reservoirs.

**Table 1:** Comparison of horizontal principal stress between prediction and testing by acoustic emission.

Number	Depth/m	Maximum horizontal stress/MPa			Minimum horizontal stress/MPa			Error (Maximum stress)/%		Error (Minimum stress)/%	
		New model	Test	Logging	New model	Test	Logging	New model	Logging	New model	Logging
S-21–1	2611.30	68.3	67.1	61.9	61.1	61.4	54.9	1.79	7.65	2.53	9.58
S-21–2	2615.27	69.1	70.1	62.8	60.3	61.6	54.3	1.43	7.35	1.36	9.88
S-21–3	2619.43	64.7	66.8	60.2	56.6	55.1	52.6	3.14	6.58	1.00	4.54
S-21–4	2628.22	62.3	67.4	60.9	48.7	49.1	46.6	7.57	9.62	0.81	5.67
S-21–5	2633.36	54.7	52.3	45.3	49.3	48.2	42.9	3.06	9.98	1.87	9.90
S-21–6	2638.41	54.1	56.2	58.6	49.7	49.9	53.1	3.74	4.10	0.40	6.03
S-21–7	2643.19	57.8	56.1	60.3	54.6	55.8	57.2	2.28	6.51	2.15	2.55
S-21–8	2650.08	55.6	56.8	59.7	50.3	51.9	53.8	3.87	4.86	3.08	3.56
S-37–1	2999.31	53.7	52.3	49.9	49.2	48.5	46.3	3.06	4.58	1.44	4.54
S-37–2	3002.21	62.5	60.7	63.9	56.1	53.6	55.8	2.28	5.01	3.02	3.56
S-37–3	3013.17	66.9	62.1	58.3	62.2	59.1	55.2	2.10	6.12	2.15	6.60
S-37–4	3016.26	65.7	62.3	60.1	60.9	59.9	53.1	2.84	3.53	0.59	9.93
S-37–5	3021.65	66.1	60.9	62.8	58.7	57.1	58.7	3.19	3.03	0.75	2.83
S-37–6	3027.15	63.9	61.1	59.6	54.5	51.5	55.8	2.35	2.51	1.98	7.71
S-37–7	3033.19	63.7	60.3	60.1	55.1	52.3	53.3	1.91	0.33	2.39	1.88
S-37–8	3039.26	64.9	61.8	63.7	56.3	51.7	54.1	1.74	2.99	2.71	4.44

**Table 2:** Comparison of Yong’s modulus between prediction and test.

Number	Depth/m	Young’s modulus/GPa			Error/%	
		New model	Test	Logging	New model	Logging
S-21–1	2611.30	15.3	17.2	13.8	8.10	9.96
S-21–2	2615.27	16.7	18.5	19.1	7.73	3.15
S-21–3	2619.43	13.1	15.2	14.6	8.81	3.95
S-21–4	2628.22	17.5	15.9	18.3	8.14	8.31
S-21–5	2633.36	19.3	17.8	20.5	7.78	9.17
S-21–6	2638.41	20.3	18.6	21.7	8.37	14.3
S-21–7	2643.19	22.7	20.3	23.9	10.6	15.1
S-21–8	2650.08	23.5	26.2	22.7	10.3	13.4
S-37–1	2999.31	26.6	29.3	25.3	9.22	14.3
S-37–2	3002.21	25.7	27.5	23.8	6.55	13.6
S-37–3	3013.17	23.8	21.5	22.6	9.66	4.88
S-37–4	3016.26	26.1	24.7	25.9	5.36	4.63
S-37–5	3021.65	27.3	23.7	25.1	13.2	5.58
S-37–6	3027.15	25.6	23.9	24.3	6.64	1.65
S-37–7	3033.19	26.9	23.2	25.2	13.8	7.94
S-37–8	3039.26	21.7	19.9	20.2	8.30	1.50

### Determination of in-situ stress direction

**Figure 13:**
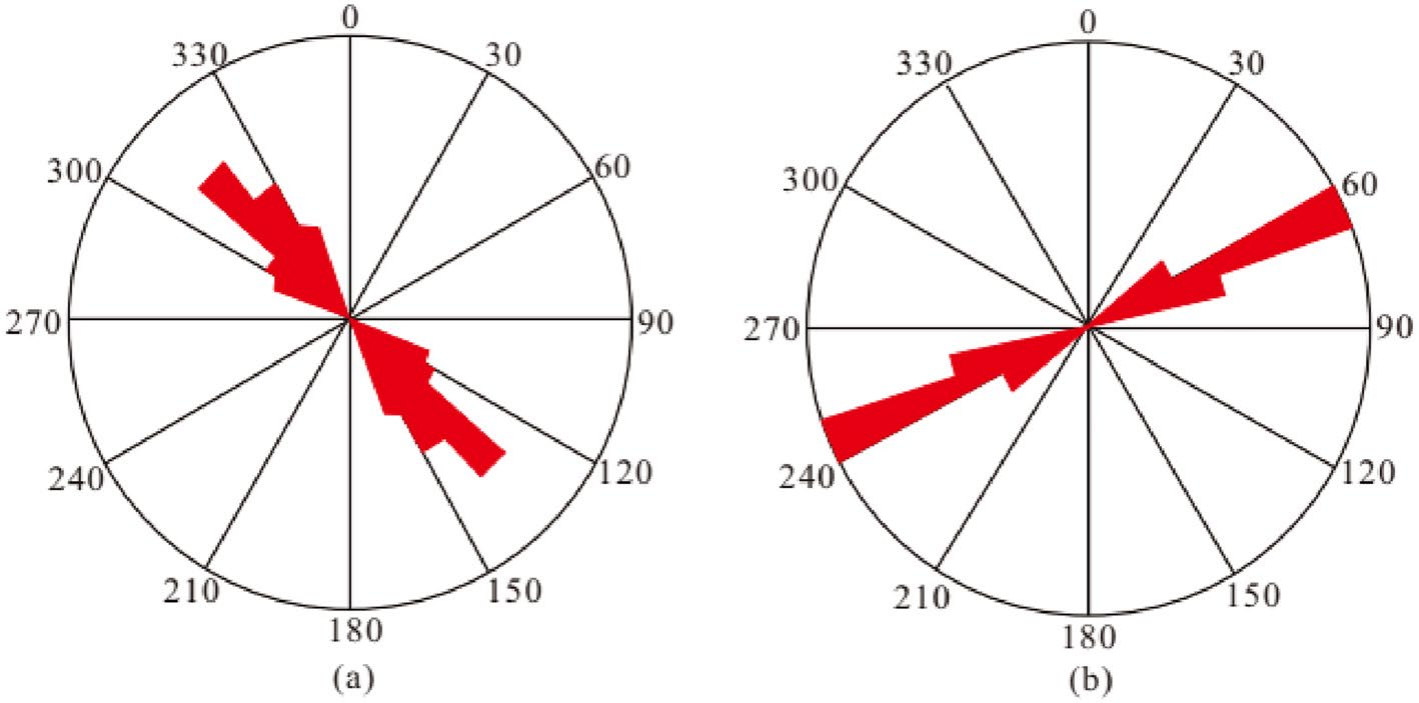
Direction of induced fracture (**a**) and long axis of elliptical hole (**b**).

According to the strike of induced fracture, the minimum horizontal principal stress direction was judged to be NE 48°, and the minimum horizontal principal stress direction was judged to be NE 63° in the long axis direction of ellipse wellbore (Fig. 13), reflecting that the maximum horizontal principal stress direction in this area was NW-SE159°. The downhole micro seismic monitoring results in the studied area showed that the artificial fracture extended in NW–SE 145°-178°, average 151°(Table 3).

**Table 3:** Results of monitoring fractures with micro seismic.

Well	J-10	J-13	J-18	J-27	J-171	J-30	J-22
Fracture height/m	49	58	51	56	53	76	57
Fracture length/m	236	251	318	269	388	231	257
Fracture direction/$^\circ$	$\frac{141-163}{156}$	$\frac{143-159}{150}$	$\frac{139-158}{146}$	$\frac{140-159}{152}$	$\frac{144-168}{158}$	$\frac{141-170}{159}$	$\frac{135-167}{151}$

**Figure 14:**
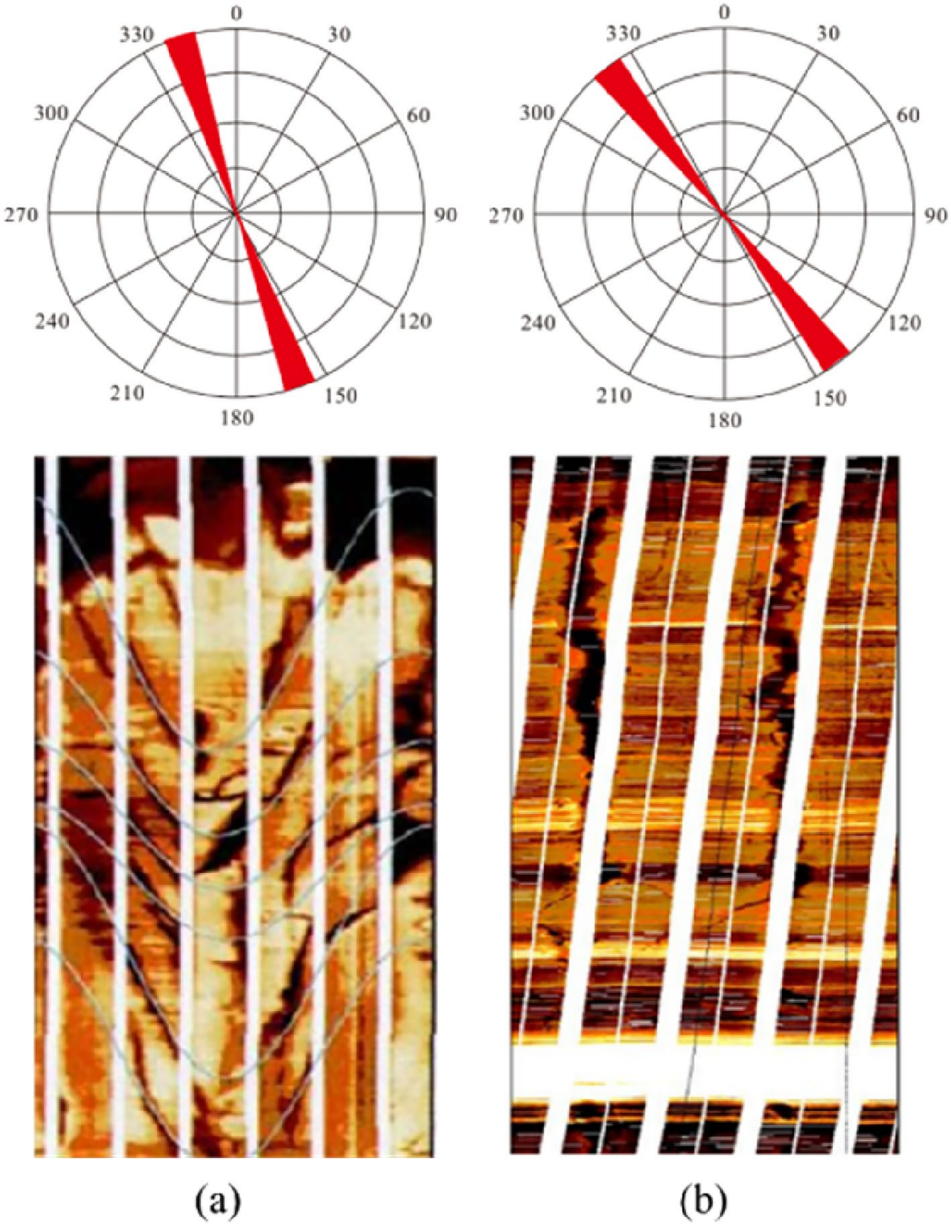
Imaging logging of pressure fracture in well J-37 and well J-21. (**a**) Pressure fracture in well J-37, 3033-3037 m, with fracture strike NE152°. (**b**) Pressure fracture in well J-21, 3011-3015 m, with fracture strike NE148°.

It can be seen from Fig. 14 that the fracture direction in the studied area is in good consistency with the current in-situ stress direction, which is conducive to the secondary reconstruction of natural fractures and keeps good opening of fractures. According to the analysis of wave velocity anisotropy and paleomagnetic data, the maximum horizontal principal stress direction in the study area is near NW-SE144°-159°. Therefore, fractures along this direction are dominant fractures in the study area.

## Discussion

### Applicability of new method

The new method proposed in this paper makes full use of the borehole deformation characteristics caused by in-situ stress, deduces the horizontal principal stress formula expressed by parameters of elliptic borehole shape and Young's modulus, and derives the Young's modulus formula expressed by drilling parameters. As the structure parameters of elliptic borehole could be measured with dual caliper logging and drilling parameters could also be acquired in real time, it does not cost much more time and expense than hydraulic fracturing. Additionally, it could also predict the in-situ stress in the deep formation(> 2000 m), breaking through limitation of measuring depth of existing methods. Therefore, the innovation of new method includes two aspects: (1) Based on elastic theory, proposing the quasi-ellipse characteristic model of borehole, subsequently, constructing the in-situ stress prediction model based on borehole deformation; (2) Performing the force analysis of diamond bit during drilling, and deriving the continuous prediction formula of elastic modulus based on weight on bit (WOB) and torque parameters. In vertical wells, the shape of the borehole is closely related to the horizontal principal stress, which can be confirmed by the elliptical caving of the borehole wall during drilling. Currently, no studies on the inverse calculation of horizontal principal stress by studying the deformation of borehole structure have been conducted. Theoretical derivation demonstrates that the shape structure of borehole under horizontal principal stress is elliptical, moreover principal stress is related to Young’s modulus and structure parameters of elliptical borehole. Based on this mathematical theory, the maximum and minimum horizontal principal stress could be achieved by accurately measuring the shape parameters of elliptical borehole, namely the long and short axes. Additionally, predicting Young’s modulus with drilling parameters has never been studied. The results show that prediction of Young’s modulus with drilling method could meet the precision of actual engineering. Consequently, above cases show that the errors of predicted horizontal principal stress prediction with new method proposed in this paper are 3.21%, 2.98%, respectively. These errors meet the requirements of engineering. However, the corresponding errors with logging model are 5.30%,5.83% respectively. Obviously, the error of new method is lower than that of logging model. Although logging method is still commonly applied in oil fields, the structural stress coefficient (${\beta }_{1},{\beta }_{2}$) and Biot coefficient ($\alpha$) in this method are empirical coefficients ^30^. Additionally, these coefficients are calculated by some data points randomly, certain human error exists in this method. Moreover, the logging model is established based on the specific geological characteristics, and constrained by certain geological condition. Therefore, the new prediction method based on elliptical deformation of borehole is more precise, practical and innovative than logging method.

### Impacting factors of new method precision

Accurately determining the major and minor axes of the quasi-ellipse is the primary key factor of the new method for in-situ stress prediction. According to Eq. (13), it can be seen that the in-situ stress measurement results are related to the elastic modulus of rock, the quasi-ellipse long semi-axis *A*, quasi-ellipse short semi-axis *B*, as well as the initial borehole radius *a*. It can be seen from laboratory measurement and prediction results, the variation difference of elastic modulus of Lucaogou Formation is small, which has little influence on the accuracy of in-situ stress prediction, while the quasi-ellipse long semi-axis *A* and short semi-axis *B* can be measured in the field. Therefore, on the premise of ensuring the measurement accuracy, the main error of in-situ stress comes from the value of the initial radius *a* of the borehole. The relative error ${\tau }_{0}$ of the predicted stress and the principal stress can be deduced by theoretical deduction, seen as following Eq. 29:

29 $$\tau_{0} = \frac{ - \tau }{{1 + \tau }}\left( {\frac{E}{{2\sigma_{H} }} + 1} \right)$$

It can be seen from Eq. (29) that the relation between the relative error ${\tau }_{0}$ of the predicted stress and the principal stress is as follows: when the relative error $\tau$ of the wellbore diameter remains unchanged, the greater the principal stress ${\sigma }_{H}$, the smaller the relative error ${\tau }_{0}$ of the predicted stress; when the principal stress ${\sigma }_{H}$ remains unchanged, the larger the relative error $\tau$ of the wellbore diameter, the larger the relative error ${\tau }_{0}$ of the predicted stress.

According to the above error analysis, certain applicable conditions and operating points in the process of in-situ stress testing based on wellbore deformation analysis exist as follows:(1) The quality of wellbore is strictly controlled, especially the wellbore diameter error; (2) New method is more suitable for wells with larger diameter, as the diameter deformation of wellbore with large diameter is larger than that with small wellbore diameter under the same conditions;(3) New method is more suitable for deeper drilling, as the in-situ stress in deep strata is usually larger than that near the surface, and the wellbore diameter deformation under in-situ stress is larger.

## Conclusion

1. Based on the theoretical derivation of elasticity and classical rock mechanics theory, it can be known that the geometric form of the wellbore under the action of plane two-dimensional stress is quasi-ellipse, in addition the quantitative relationship between horizontal principal stress and quasi-ellipse geometric parameters is established. The statistical analysis of wellbore diameter parameters can achieve the quantitative characterization of quasi-ellipse geometric parameters.
2. The elastic modulus prediction model based on drilling parameters is deduced, making the continuous prediction of elastic modulus based on drilling parameters applicable, thereby constructing a measurable and continuous in-situ stress prediction model.
3. Compared with in-situ stress measurement with acoustic emission, the prediction error is less than 4%, which meets the actual precision requirements. The prediction results show that the minimum horizontal principal stress of Lucaogou Formation is 51.1–62.7 MPa, and the maximum horizontal principal stress is 58.9–69.1 MPa, respectively. The maximum horizontal principal stress direction is NW-SE159°. The average difference coefficient of horizontal principal stress is 0.69, and the brittleness of reservoir rock is small.

## Data Availability

The datasets generated and/or analyzed during the current study are not publicly available due to the requirements of laboratory but are available from the corresponding author on reasonable request.
